# Infectiousness of places – Impact of multiscale human activity places in the transmission of COVID-19

**DOI:** 10.1038/s42949-022-00074-w

**Published:** 2022-11-03

**Authors:** Lun Liu, Hui Wang, Zhu Zhang, Weiyi Zhang, Shengsheng Zhuang, Shenhao Wang, Elisabete A. Silva, Tingmiao Lv, Chi On Chio, Yifan Wang, Rina Dao, Chuchang Tang, On Ieng Ao-Ieong

**Affiliations:** 1 School of Government, Peking University, Beijing, China; 2 Institute of Public Governance, Peking University, Beijing, China; 3 School of Architecture, Tsinghua University, Beijing, China; 4 Peking Union Medical College, Beijing, China; 5 Department of Urban and Regional Planning, University of Florida, Gainesville, FL USA; 6 Department of Urban Studies and Planning, Massachusetts Institute of Technology, Cambridge, MA USA; 7 Media Lab, Massachusetts Institute of Technology, Cambridge, MA USA; 8 Department of Land Economy, University of Cambridge, Cambridge, UK; 9 Lab of Interdisciplinary Spatial Analysis, University of Cambridge, Cambridge, UK

**Keywords:** Geography, Economics, Risk factors

## Abstract

COVID-19 raises attention to epidemic transmission in various places. This study analyzes the transmission risks associated with human activity places at multiple scales, including different types of settlements and eleven types of specific establishments (restaurants, bars, etc.), using COVID-19 data in 906 urban areas across four continents. Through a difference-in-difference approach, we identify the causal effects of activities at various places on epidemic transmission. We find that at the micro-scale, though the transmission risks at different establishments differ across countries, sports, entertainment, and catering establishments are generally more infectious. At the macro-scale, contradicting common beliefs, it is consistent across countries that transmission does not increase with settlement size and density. It is also consistent that specific establishments play a lesser role in transmission in larger settlements, suggesting more transmission happening elsewhere. These findings contribute to building a system of knowledge on the linkage between places, human activities, and disease transmission.

## Introduction

Humans continue to migrate to large, dense urban settlements in the past century. The consequent growth of cities brings benefits such as economies of scale and knowledge spillovers, but also increases the vulnerability of daily life to risks related to people’s agglomeration and interaction, such as congestion, crime, and infectious disease^1,2^, for which COVID-19 is a prominent example. In the spread of these risks, places containing different activities are key risk units that link physical environments, human activities, and risk factors. To understand the impact of different places in risk transmission would be important for the science and practice in enhancing the resilience and life quality of human settlements.

The strike of COVID-19 raises the concern about the epidemiological risks of places, which, however is seldom evaluated. In theory, different types of activity places could lead to different chances of virus transmission. At the macro-scale, dense settlements lead to physical proximity among residents, and large settlements connect more people—both might generate more contacts and increase the dissemination of infectious diseases^3–5^. At the micro-scale, different types of establishments, such as restaurants, museums, and sports fields, are also likely to generate different chances of virus transmission, influenced by the contacts made through corresponding activities and the physical environments. This paper, therefore, aims to quantify the virus transmission risks associated with different settlement characteristics at the macro-scale and establishments at the micro-scale as well as their interactions, to build a system of knowledge on the infectiousness of human activity places and inform relevant policy-making.

Though the risk of virus transmission at different types of places can be evaluated with mechanistic modeling^6,7^, it is difficult to find data to meaningfully calibrate the strengths of different human interactions in the simulation. Alternatively, we take advantage of the natural experiments provided by the diverse government interventions, including the closure of many activity places across regions and countries in COVID-19 and examine the role of places in virus transmission with natural experimental methods. Although there have been a body of research using these empirical data to estimate the efficacy of government interventions in COVID-19, our work is more fine-grained in the types of activity places examined^8–18^. In this work, we examine the impacts of two macro-scale place characteristics, population size and density, which have been found to affect many social quantities^5,19^, and eleven common micro-scale establishments, that are schools, childcare centers, offices, non-essential retails, restaurants, bars, entertainment venues, cultural venues, religious venues, indoor sports venues and outdoor sports grounds (detailed descriptions in Supplementary Table 1). We use four countries from four continents as study cases, which are Japan in Asia, the United Kingdom in Europe, the United States in North America, and Brazil in South America (Supplementary Figs. 1–4). The four countries are diverse in settlements’ spatial form, lifestyle, culture, and government actions in COVID-19, which could enhance the generalizability of our findings.

We employ a natural experiment-based econometric approach called difference-in-differences (DiD), which is widely used in examining the causal relationship in social sciences and estimates the causal impact of treatment through differences in treatment timing in different units^20^. To be more specific, for estimating the impact of a group of establishments in virus transmission, the DiD method subtracts the course of the epidemic in spatial units where that group of establishments get closed or reopened from the epidemic course in spatial units where the status of the same establishments remain unchanged, assuming that the epidemic in the two groups should move in parallel trend absence of the change. By subtracting the trends, this method can rule out the influence of simultaneous behavioral changes shared by all spatial units. Simultaneous behavioral changes could happen when people started to be more cautionary as an intervention got implemented, driven by the gravity of the pandemic or the signaling effect of the intervention, resulting in the overestimation of intervention effects^10^. The DiD method can subtract out the common behavioral changes in a country, thus alleviating the problem of overestimation^21^. The choice of spatial units in each sample country is based on two criteria: first, infection data and other socioeconomic data are available for the spatial units; and second, the spatial units are as close as possible to the spatial extents of settlements (a continuously built-up area). Spatial units with a population larger than 100,000 are taken as samples, as smaller units may not have enough infection cases to produce reliable estimates. These criteria led to 45 spatial units in Japan, 234 in the United Kingdom, 308 in the United States, and 319 in Brazil after cleaning missing data (detailed explanations on the choice of spatial units in Supplementary Methods).

**Fig. 1:**
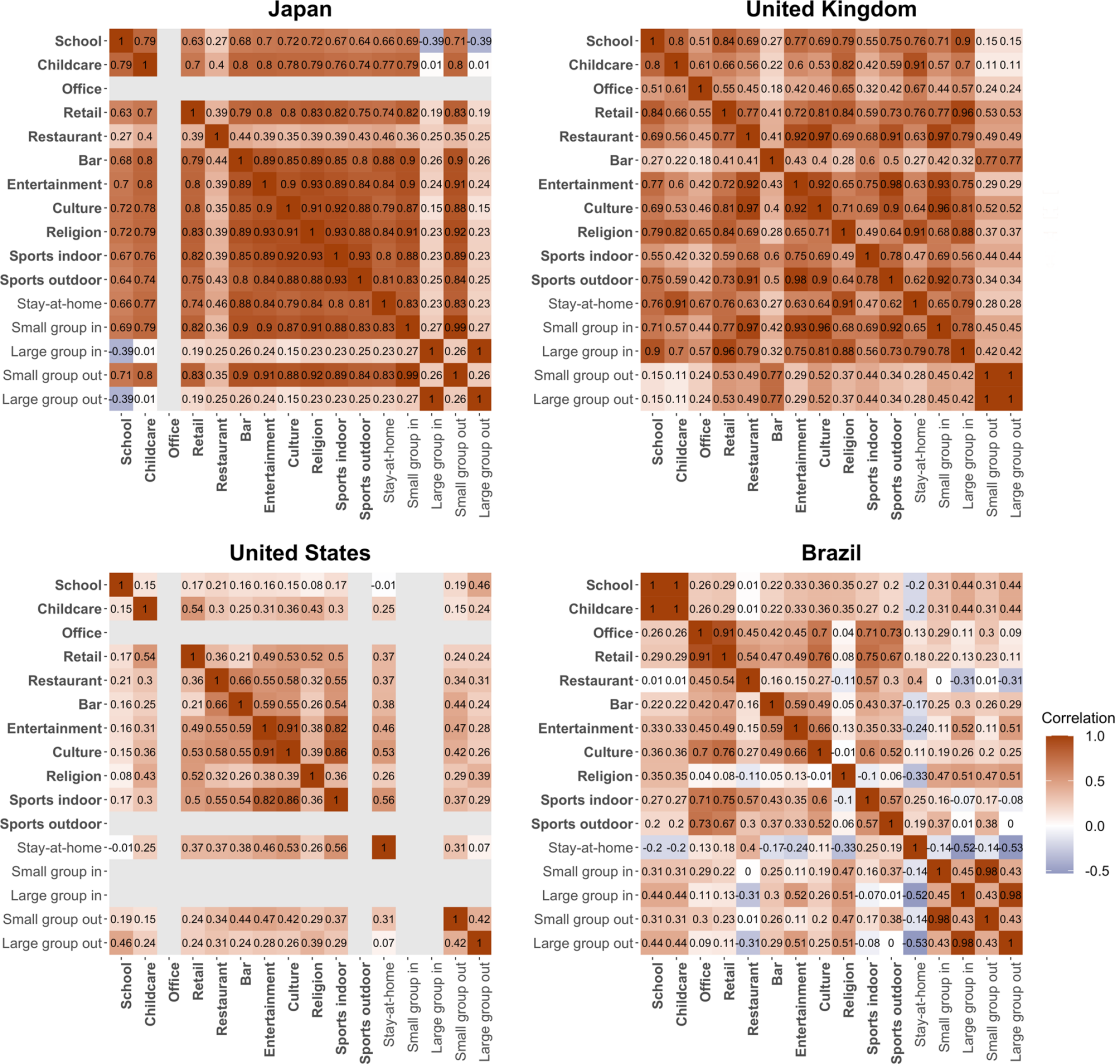
Correlation between the status of establishments and other government interventions. The matrices show pairwise Kendall’s correlation coefficients between the status of establishments and other government interventions across the spatial units in each country during the study period. Bold texts indicate the establishments and regular texts are the other government interventions that we control for. Gray lines indicate that information on the corresponding intervention is missing in the country. The correlation coefficients are estimated based on samples excluding unit-day observations where the coefficient of variance for *R*_*t*_ estimate is larger than 0.3 (suggesting unreliable estimates), which are 2400 in Japan, 24,285 in the United Kingdom, 41,752 in the United States, and 34,812 in Brazil.

We start by evaluating the risks of virus transmission at different types of establishments, by estimating the causal impacts of establishment closures on the course of the epidemic. This is implemented by modeling the relationship between instantaneous reproduction numbers (*R*_t_) in the spatial units and the status of various establishments, controlling for other interventions (stay-at-home orders and gathering bans). We estimate separate models for each country to allow for heterogeneous infection risks at various establishments in different countries, considering cross-country differences in lifestyle, culture, urban form, etc. Correlation analysis shows that Kendall’s correlation coefficients between the status of establishments are mostly lower than 0.8 in our data set, despite that in some cases, governments close or reopen multiple types of establishments together (Fig. 1), providing at least 180 unit-day differences between the status of any pair of establishments. We also verify that the estimates are not sensitive to removing establishment status variables, suggesting manageable collinearity (Supplementary Methods). The estimation is implemented through a two-way fixed effect model with fixed effects of days and spatial units, which is a widely used modeling method to implement DiD analysis^22^.

To perform the analysis, we combine data from a variety of sources, including COVID-19 infection case data, government intervention data, and socioeconomic characteristics of spatial units (see Supplementary Methods for a detailed description of data sources). We use data from the first pandemic wave, that is, from March to August 2020, since there could be more factors potentially biasing the analysis in later periods of the pandemic, including lockdown fatigue, virus variants, vaccination, etc.^9^.

## Results

### Infection risks at micro-scale establishments

**Fig. 2:**
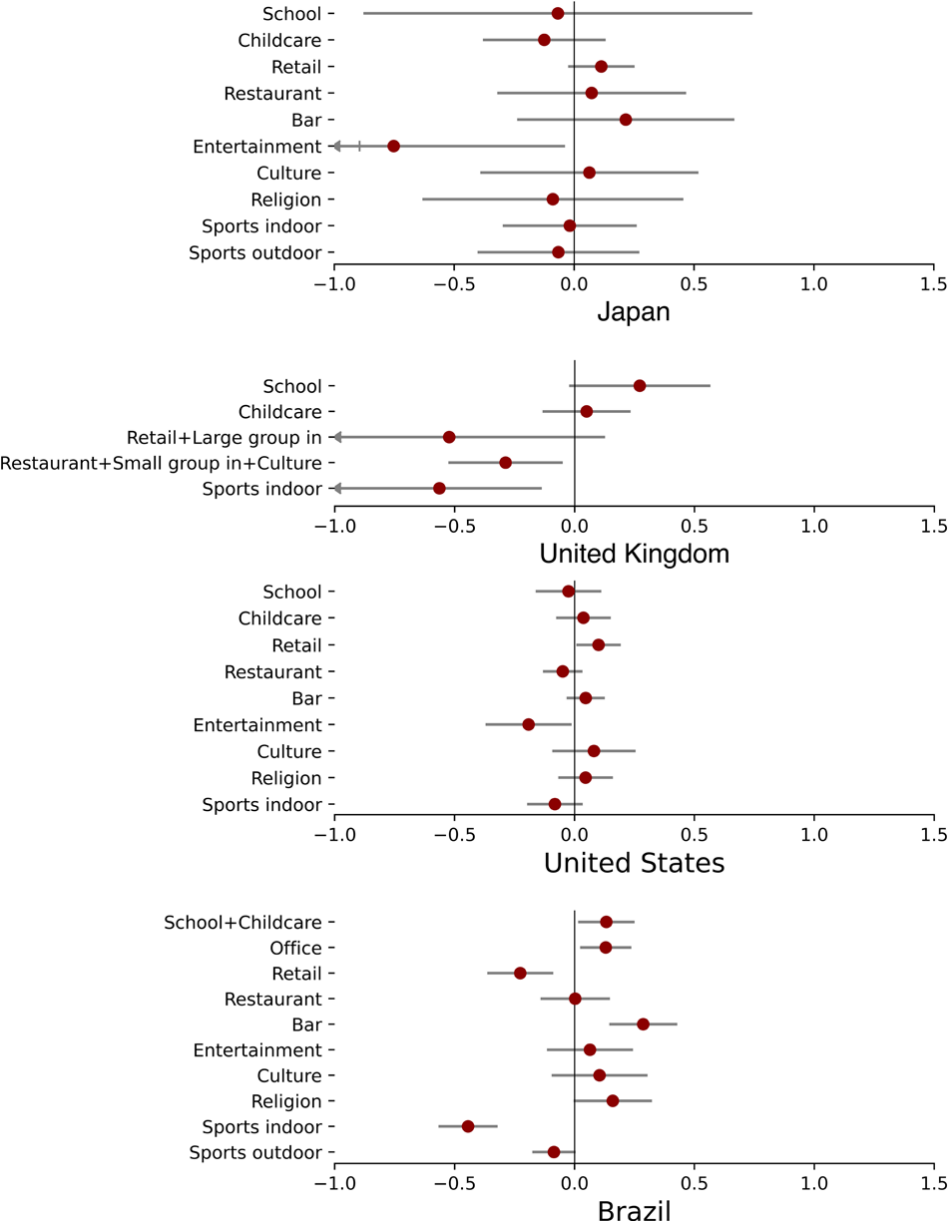
Estimated impacts of closing individual types of establishments. The numbers are direct model outputs on the relationship between establishment status (0, 0.5, or 1) and *Δlog(R*_*t*_*)*. Full results are presented in Supplementary Table 2. The error bars represent 95% confidence intervals.

The DiD analysis provides estimates on the percentage reduction in *R*_t_ caused by closing each type of establishment (computed from direct model outputs as 1-*e*^x^, where *x* denotes direct model outputs shown in Fig. 2). The reductions can be further interpreted as the proportions of total infections related to the respective type of establishments, which could happen through human interactions either inside these places or on the way to these places. Closures of establishments that show a statistically significant impact on reducing *R*_t_ in each country are (with a 95% confidence interval): entertainment venues (53%, 4 to 77%) in Japan; restaurants and cultural venues (combined with indoor gathering ban whose effect is inseparable, 25%, 5 to 41%) and indoor sports venues (43%, 13 to 63%) in the United Kingdom; entertainment venues (17%, 1 to 31%) in the United States; and non-essential retails (20%, 9 to 31%) and indoor sports venues (36%, 27 to 43%) in Brazil (Fig. 2, full model results in Supplementary Table 2). These results reflect the establishments with the largest epidemiological risks in each country.

Most of the establishment status variables satisfy the parallel trend assumption, meaning that the estimates are not biased by potentially different pre-trends of *R*_t_ in areas that close or reopen a group of establishments and those that do not (detailed methodology and results of the parallel trend test in Supplementary Methods and Supplementary Table 3). The estimates are also generally robust to a number of alternative settings in the analysis, including withholding spatial units from the sample and increasing or decreasing variables in the model, suggesting that they are not likely to be affected by individual influential spatial units and the correlation among variables (Supplementary Figs. 5, 6, detailed methodology and results in Supplementary Methods).

**Fig. 3:**
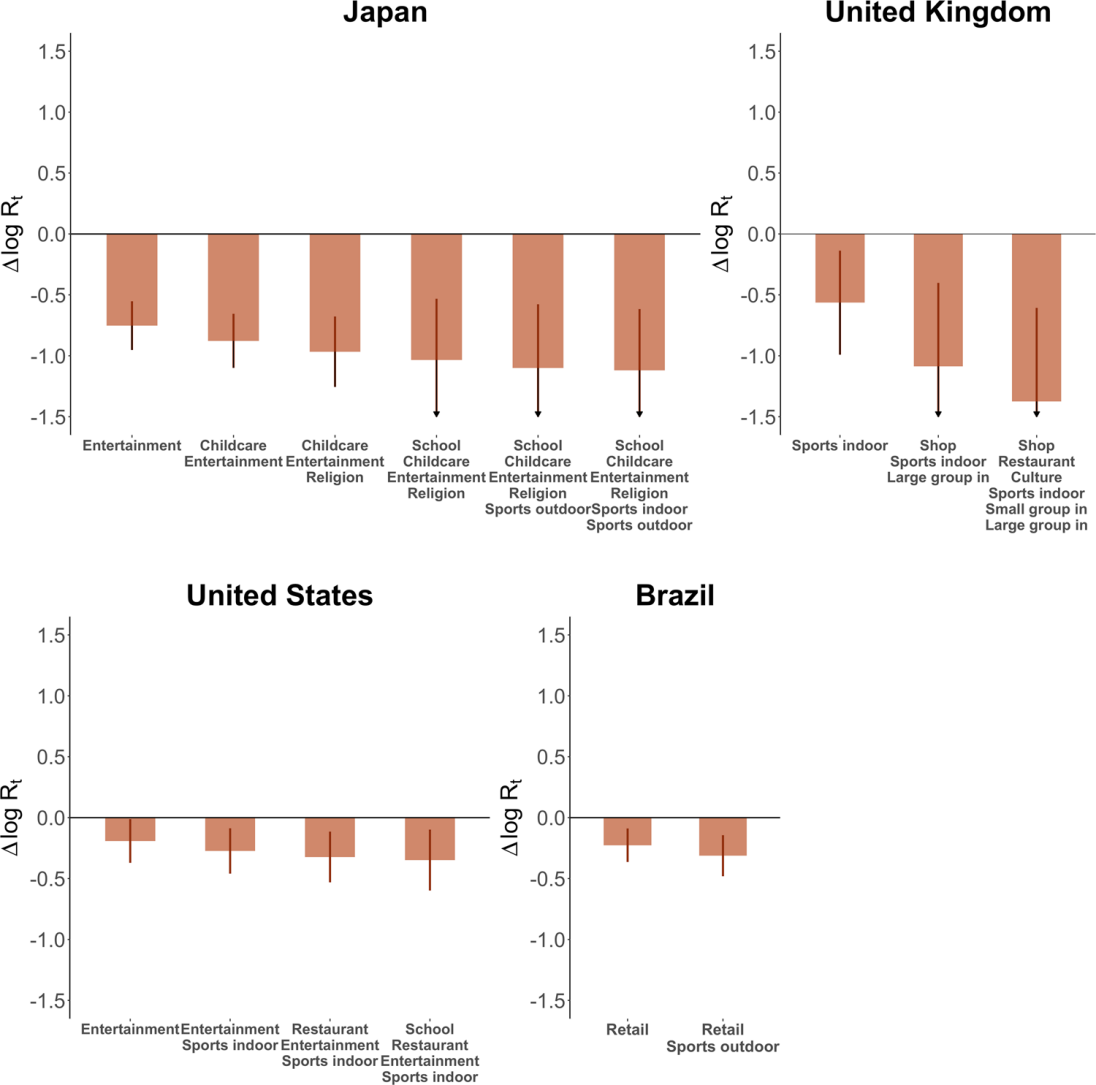
Maximum joint impacts of closing a number of establishments. We show the maximum impacts that could be achieved by closing a given number of establishment types, till the maximum joint impacts are produced. The error bars represent 95% confidence intervals.

Considering that epidemic response plans often need to identify a set of establishments with the largest combined impact, we further estimate the joint impacts of all possible combinations of establishments in each country based on the previous results. The full results can be found in the repository of this project (see Code Availability). Here we present the maximum reduction in *R*_t_ that can be achieved by closing a given number of establishments (Fig. 3). Our analysis suggests that the largest reductions in *R*_t_ are achieved by closing two to six types of establishments, while more closures do not further bring reproduction numbers down. Governments could resort to this kind of analysis when making cost-effective intervention strategies.

### Infection risks at macro-scale settlements

The fixed effects of spatial units in the two-way fixed effect model estimated in the previous step can be interpreted as the intrinsic speed of virus transmission in each spatial unit absence of any spontaneous or compulsory behavioral changes. This fixed effect could be a combined outcome of settlements’ spatial (such as size and density) and socioeconomic characteristics, such as the age, ethnicity, and wealth of residents. Based on this, we estimate the impacts of settlement size and density on this intrinsic speed of virus transmission using linear regression with the unit fixed effects as the dependent variable and settlement socioeconomic characteristics as control variables.

The impacts of settlements’ population size and density are fairly consistent across the four countries (Table 1). Population size is negatively correlated with spatial unit’s fixed effect on *R*_t_ and this is statistically significant in three of the four countries, where the effect size ranges between 2.0% (1.1 to 3.2%) to 4.9% (2.3 to 7.5%) reduction of *R*_t_ per million increase of population. The impact of density is less clear, yet none of the estimates is positively significant, as suggested by the common beliefs mentioned in the introduction. These results contradict the impression that large and densely populated cities tend to be epicenters and suggest that in terms of the reproduction number, large and dense cities are not riskier, but even less. Explanations for the negative relationship between settlement size and *R*_t_ might include better health infrastructures in large cities and people’s stronger awareness of the risk, thus more cautious behavior^23,24^. Nonetheless, more data is needed to testify these possible explanations.

**Table 1:** Impact of settlement characteristics on the intrinsic speed of virus spread.

Characteristics	Japan	United Kingdom	United States	Brazil
Size	−0.118 (0.113)	−0.029** (0.0111)	−0.0219*** (0.00531)	−0.0506*** (0.0139)
Density	−0.203 (0.392)	−0.00108 (0.00539)	0.00453 (0.0201)	−0.0184** (0.00648)
Proportion of elderly population	−0.102 (2.29)	−0.0325 (0.2)	0.535*** (0.155)	−0.228 (0.387)
Proportion of Black	–	−0.105 (0.425)	−0.000726 (0.000542)	–
Proportion of Asian	–	0.162 (0.124)	0.00304* (0.00148)	–
Personal income	−0.125 (0.105)	–	−0.00686*** (0.000926)	0.105* (0.0435)
GDP per capita	0.00494 (0.00855)	−0.00123* (0.000592)	0.002** (0.000635)	−0.000228 (0.000374)
(Intercept)	0.781 (1.68)	−0.158** (0.0514)	0.101** (0.0386)	0.217*** (0.0463)
Observations	44	234	307	319
R-squared	0.294	0.0622	0.345	0.125
Adjusted R-squared	0.198	0.0373	0.329	0.111

Results in this table are based on samples excluding outliers (Shimane in Japan, Mendip in the United Kingdom, and Indianapolis-Carmel-Anderson, Pittsfield, and San Angelo in the United States), so that the residuals are normally distributed (Shapiro–Wilk test *p* > 0.05). Results on full samples are very similar (shown in Supplementary Table 4). The variance inflation factors are all below 7.

*p < 0.05, **p < 0.01, ***p < 0.001

### Varying risks by the interaction between the two scales

We also examine the interaction between macro-scale settlement characteristics and micro-scale establishments in the transmission of COVID-19, since the activity pattern of residents in different types of settlements could be different, leading to the heterogeneous distribution of infection risks. To do this, we re-estimate the maximum joint effects of establishment closures on separate samples of relatively large and small, and high-and low-density spatial units. The high/low samples are split by the median population size (174,980 people) and density (681 people per squared kilometer) of all sample spatial units, except for Japan, where the population size and density are generally much higher so we use the median of its own (314,082 people and 5671 people per squared kilometer, respectively). More details on the specification of the models and sensitivity tests can be found in Methods and Supplementary Methods.

**Fig. 4:**
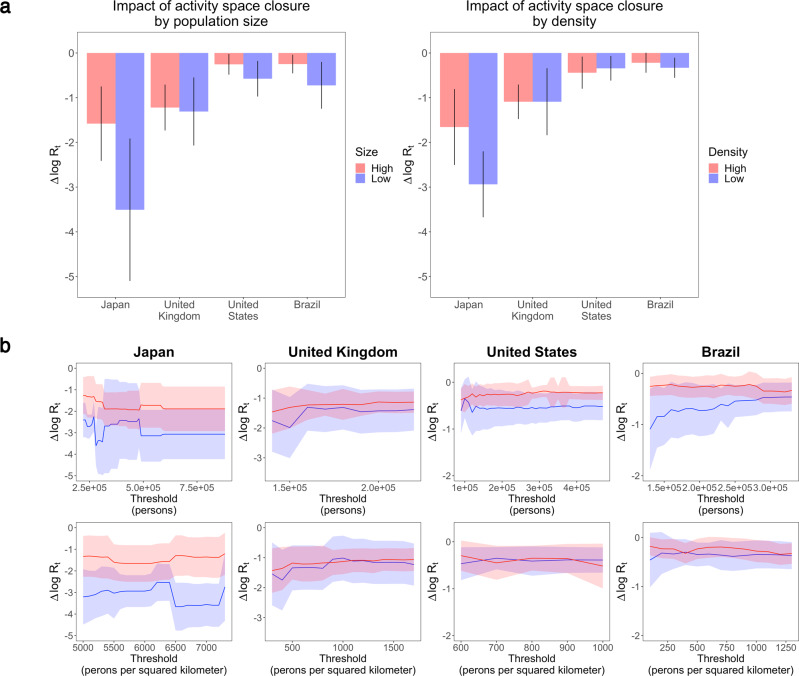
Impact of establishment closures in settlements with different population size and density. **a** Comparison of the impacts in relatively large and small, and high and low density settlements, split by the median settlement population size and density in the sample. **b** Comparison of the impacts using different population size and density cut-off values. The error bars and ribbons represent 95% confidence intervals.

The comparisons are remarkably consistent across the four countries in terms of the interaction with settlement size—the impacts of establishment closures are larger in relatively small settlements, reflecting a higher share of infections accounted for by specific establishments in smaller settlements (Fig. 4a). In other words, a larger proportion of infections are related to general public spaces in large settlements, which might include streets, public transits, etc.^25^. The disparity in the proportion of infections accounted by establishments ranges between 3 and 18%. The impacts are also larger in relatively low-density settlements in Japan and Brazil, and the impacts are close to each other in the United Kingdom and the United States. Nonetheless, no common pattern is observed for individual types of establishments. This could be because there are many variations in terms of the physical conditions and social interactions at these activity places in different countries.

To test whether the results hold when the population and density thresholds change, we repeat the analysis with a series of cut-off values between the first and third quantiles of population size and density in each country. The results are generally stable regardless of the threshold used to split the samples, and are particularly consistent in terms of settlement size: the proportion of infections accounted for by various establishments are always bigger in relatively small settlements than in large settlements in all sample countries (Fig. 4b). Similar pattern also exists with regard to settlement density in Japan and less prominently in Brazil. The pattern is also robust with alternative indicators of settlement size and density (Supplementary Fig. 7).

## Discussion

Our analysis quantifies the virus transmission risks at different types of human activity places using evidence from the COVID-19 pandemic. The work contributes to emerging literature on the health and resilience of human settlements and takes the initial steps towards developing a system of knowledge on the infectiousness of activity places^26–28^. The results can inform minimal impact resilience plans for not only the ongoing COVID-19 but also future public health crisis, as well as long-term strategy making in reducing the epidemiological risks in human settlements.

Data from COVID-19 suggests that closing various establishments could, at most, reduce *R*_t_ by 27 to 75% in the four sample countries; in other words, activities at these establishments lead to 27 to 75% of all infections. The magnitudes of the impacts are heterogeneous across countries, which could be affected by the behaviors and interactions at relevant places, socioeconomic profiles of the visitors, physical conditions of relevant spaces, as well as the level of enforcement. For example, the small effect sizes in the United States and Brazil might be related to loose enforcement and non-compliance, thus might not reflect the true impact of establishments in virus transmission in the two countries^29,30^. The heterogeneous results suggest that while it sounds strong to draw general conclusions on the infection risks of activity places, such conclusions could run the risk of over-simplification and diverge from the reality for individual countries. Despite the heterogeneity, it is common in the four sample countries that the closures of essential activity places, including schools, childcare centers, and offices, do not demonstrate statistically significant effects in reducing *R*_t_ while closing certain non-essential activity places, including sports fields, entertainment venues, and restaurants, tend to be more effective. Resilience plans for future public health crisis could first consider interventions targeted at these places, which is likely to be more cost-effective.

A few issues regarding our effect estimates should be noted. Our effect estimates are smaller than many existing studies on COVID-19 intervention effects, e.g. we do not find a significant effect of school closure in reducing *R*_t_. On one hand, it could be because the DiD approach is able to partly rule out the impact of increased self-protection happening simultaneously with interventions. On the other hand, it could also be a limitation of our data as the data are from only four countries and from the early phase when interventions were implemented close to each other. As a result, our effect estimates of activity place closures are associated with large confidence intervals, which may actually contain the estimates from other studies. Besides, for Brazil, our estimates show that the closures of a few establishments increase *R*_t_. A possible reason is that the enforcement level of venue closures might be loose in Brazil as there were conflicting opinions towards the interventions (Supplementary Notes). Another explanation could be related to the wide existence of ghettos in Brazilian cities, where the high density might increase the chance of transmission when people stay more at home. And for the UK, larger variations are associated with the effect estimates, especially for retail closure (Supplementary Fig. 5). This is because there is little cross-city variation in the timing of venue closures in the UK, so the effect estimates are largely impacted by the pandemic dynamics in the few cities taking different intervention schemes. Last, the chance of virus transmission at a specific establishment could also be related to its popularity, the socioeconomic portfolio of its visitors^31^, etc. The effects we identify should be interpreted as an average of the impacts of closing individual places of a certain type.

For macro-scale settlement characteristics, our findings contradict the common belief that large and densely populated cities are more vulnerable to infectious disease^32^. This could either be because the seemingly increased connectivity and proximity among people in large and dense cities do not actually enhance the chance for virus transmission, or such an effect does exist but is offset by other positive factors, such as more healthcare resources driven by the economy of scale and more cautious behavior of people. The exact causal chain could also involve the demography, education, economy, and even partisanship in different types of settlements^24,33^, which is subject to further study. Either way, these results lend more confidence to encouraging the agglomeration of people and high-density development. It should be noted that some previous studies demonstrate a positive relationship between city size and transmission^3^. This is not necessarily contradictory to our results, since different pandemic indicators are used. For example, Hamidi et al. take infection rate as the dependent variable. Suppose that a small city and a large city have infection numbers proportional to their population size, the large city would have a higher infection rate after some time, since infections grow exponentially. Therefore, when using the infection rate as the pandemic indicator, large cities tend to have larger numbers.

The finding that the eleven types of establishments account for a smaller proportion of infections in relatively large settlements suggests that more infections take place in public spaces other than the confined areas of establishments in large settlements, which might be explained by generally longer travel distances in large settlements thus more contacts on streets, public transits, etc. It indicates that governments could rely less on closing establishments, which is economically risky, and resort to other measures to reduce infection transmission in large cities, such as contact tracing or more intense disinfection of public spaces.

Nonetheless, our findings could be affected by a number of limitations. First, besides human activities and physical environments, the virus transmission risks are also affected by the characteristics of pathogens, including the means of transmission, the susceptible population group, etc. The findings drawn from COVID-19 might apply to respiratory infectious diseases, but might not reflect the risks associated with other infectious diseases, which would further contribute to a system of knowledge on the infectiousness of places.

Second, in terms of the causal identification strategy, the DiD method requires both parallel trend and exogeneity of the treatment. While the parallel trend assumption is examined with an event-study design (Supplementary Methods), the exogeneity assumption could be challenged by unobserved confounders that affect both *R*_t_ and the closure of establishments. Though we are able to rule out a number of confounders by including a large set of government intervention variables as well as a unit and day-fixed effects, there could still be endogeneity arising from omitted unit-specific time-varying factors. For instance, a sudden outburst of infections in a hotspot may affect both governments’ interventions and local residents’ cautionary behavior, which then affects *R*_t_.

Third, since the impacts of closing different types of establishments are estimated in one model, the results could be subject to the so-called “table 2 fallacy,” which refers to that the coefficients of confounders in a model are wrongly interpreted as full causal effects while they are actually only the direct effects^34^. This problem applies if decisions to close or reopen establishments affect each other so that they become confounders. While this is possible, we suppose such a relationship should be weak since these decisions tend to be more directly affected by the trends of infections, instead of the status of other interventions.

Fourth, we assume a linear relationship between *R*_t_ and the independent variables in the entire analysis, which is a convenient assumption made by many studies on intervention effects in COVID-19^8,10,12,16,35^. However, the impact of closing one type of establishment may rely on the status of other establishments, since the corresponding activities could be complementary or substitutive to each other, leading to interacting effects. It is encouraging that studies which examine nonlinear relationships and sequence of interventions do not find significant patterns^8,36,37^, but the issue cannot be ignored.

Our work systematically examines the role of multitype and multiscale activity places in the transmission of infectious disease. Actually, public health concerns have been a key factor in shaping the planning and management of cities as early as the time of John Snow at the advent of modern cities. Our findings show that with increased human agglomeration and interaction, epidemic control no longer only involves confined areas such as hospitals or the water supply system, but also the entire urban space. Improving our knowledge of the linkage between places, human activities, and diseases would be important for long-and short-term policy-making in public health, urban resilience, and the planning of human settlement.

## Methods

### Data

We curate a data set combining daily infection cases, government interventions (including establishment closures, stay-at-home orders, and gathering bans), and the spatial, demographic and economic characteristics of the spatial units in our study, from the onset of the pandemic till August 15, 2020. The spatial units are 45 prefectures in Japan, 234 local authority districts in the United Kingdom, 308 metropolitan statistical areas in the United States, and 319 municipalities in Brazil (detailed explanations on the choice of spatial units in Supplementary Methods). Note that the prefectures (the first-level administrative division) of Japan are larger than the spatial units in other countries and contain more than one large settlement in many cases. However, infection data can only be consistently acquired at this level in Japan^37^, so it is taken as the unit of analysis. Nonetheless, we prove that the choice of spatial units would not substantially affect the results (Supplementary Methods).

The infection case data are sourced from Japan Broadcasting Corporation’s case reports, the UK government, Johns Hopkins University, and the Brazilian Ministry of Health. The timetable of government interventions is manually collected from the websites of national and state-level governments, which are the main levels of authorities making decisions on interventions. The settlement-related information is gathered from a number of official websites. More details on data sources are provided in Supplementary Methods.

### Estimating impacts of closing individual types of establishments

The causal impacts of closing individual types of establishments across all spatial units and subgroups of spatial units in a country are estimated with a two-way fixed effect model specified as follows

1 $$\log \left( {R_{c,\;i,\;t}} \right) = \beta _{{{\mathrm{c}}}}{{{\mathrm{X}}}}_{{{{\mathrm{c}}}},\;{{{\mathrm{i}}}},\;{{{\mathrm{t}}}}} + \theta _{{{\mathrm{c}}}}{{{\mathrm{Z}}}}_{{{{\mathrm{c}}}},\;{{{\mathrm{i}}}},\;{{{\mathrm{t}}}}} + \alpha _{c,\;i} + \tau _{c,\;t} + \varepsilon _{c,\;i,\;t}$$

where log(*R*_c,i,t_) is the log-transformed instantaneous reproduction number in unit *i* of country *c* on day *t*; **X**_**c,i,t**_ is a vector denoting the status of the 11 types of establishments, and **β**_**c**_ denotes the corresponding coefficients to estimate. We log-transform *R*_c,i,t_ following the practice of relevant works^10,14^, based on the plausible assumption that the reduction of *R*_c,i,t_ by the closure of establishments should be proportional to the proportion of contacts avoided instead of an absolute value, and the impacts should be smaller when *R*_c,i,t_ is already low. **Z**_**c,i,t**_ and **θ**_**c**_ denote the status of five other government interventions and their coefficients (detailed description of these interventions in Supplementary Table 1); *α*_c,i_ and *τ*_c,t_ denote the unit and time fixed effects, respectively; and *ε*_c,i,t_ denotes the error term. For the uncertainty over the parameters, we estimate robust standard errors allowing for *ε*_c,i,t_ to cluster at the unit level, to account for heterogeneity in the treatment effects^38^. If the statuses of two types of establishments are highly correlated in a country (Kendall’s correlation coefficients larger than 0.95), then they are treated as one combined type to avoid collinearity (Fig. 1).

### Estimating joint impacts of multiple establishments

The point estimates of the joint impacts are computed by summing the corresponding coefficients estimated by Eq. (1): $\mathop {\sum}\nolimits_{s \in P} {\beta _{c,\;s}}$, where *β*_c,s_ denotes the coefficient of closing establishment *s* in country *c* and *P* denotes a set of establishments. The standard errors are computed from the robust standard errors and covariances as follows

2 $${{{\mathrm{SE}}}}_{{{{\mathrm{c}}}},\;{{{\mathrm{P}}}}} = \sqrt {\mathop {\sum}\limits_{s \in P} {{{{\mathrm{SE}}}}_{{{{\mathrm{c}}}},\;{{{\mathrm{s}}}}}^2} + \mathop {\sum}\limits_{s \in P,\;s^\prime \in P,\;s \ne s^\prime } {{{{\mathrm{COV}}}}_{{{{\mathrm{c}}}},\;{{{\mathrm{s}}}},\;{{{\mathrm{s}}}}^\prime }} }$$

where SE_c,P_ denotes the standard error of the joint impacts of set *P* in country *c*; SE_c,s_ denotes the robust standard error of closing establishment *s* estimated by Eq. (1); and COV_c,s,s’_ is the covariance between the impacts of establishment *s* and *s’*.

### Estimating impacts of macro-scale settlement characteristics

We take the unit fixed effects estimated by Eq. (1), which can be interpreted as the intrinsic reproduction number in each spatial unit, and model their relationship with the size and density of settlements while controlling for the proportion of the elder population (over 65 or 60 years old depending on data availability), the proportion of Black and Asian (in the United Kingdom and the United States only), the average income of residents and the per capita gross domestic product, using simple linear regression.

3 $$\begin{array}{l}\alpha _{{{{\mathrm{c}}}},{{{\mathrm{i}}}}} = \sigma _{{{{\mathrm{c}}}},1}{{{\mathrm{DENSITY}}}}_{{{{\mathrm{c}}}},{{{\mathrm{i}}}}} + \sigma _{{{{\mathrm{c}}}},2}{{{\mathrm{POPULATION}}}}_{{{{\mathrm{c}}}},{{{\mathrm{i}}}}} + \sigma _{{{{\mathrm{c}}}},3}{{{\mathrm{OLD}}}}_{{{{\mathrm{c}}}},{{{\mathrm{i}}}}} + \sigma _{{{{\mathrm{c}}}},4}{{{\mathrm{BLACK}}}}_{{{{\mathrm{c}}}},{{{\mathrm{i}}}}} \\\qquad+\, \sigma _{{{{\mathrm{c}}}},5}{{{\mathrm{ASIAN}}}}_{{{{\mathrm{c}}}},{{{\mathrm{i}}}}} + \sigma _{{{{\mathrm{c}}}},6}{{{\mathrm{INCOME}}}}_{{{{\mathrm{c}}}},{{{\mathrm{i}}}}} + \sigma _{{{{\mathrm{c}}}},7}{{{\mathrm{GDP}}}}_{{{{\mathrm{c}}}},{{{\mathrm{i}}}}} + \psi _{{{\mathrm{c}}}} + \xi _{{{{\mathrm{c}}}},{{{\mathrm{i}}}}}\end{array}$$

where DENSITY_c,i_, POPULATION_c,i_, OLD_c,i_, BLACK_c,i_, ASIAN_c,i_, INCOME_c,i_, and GDP_c,i_ denote the density, population size, proportion of the elder population, proportion of Black, proportion of Asian, residents’ income and per capita gross domestic product in unit *i*; *σ*_c,1_ to *σ*_c,7_ are their coefficients; *ψ*_c_ is the constant and *ξ*_c,i_ is the error term.

## Supplementary information

Supplementary Information

## Data Availability

The datasets used in the study are available in the Github repository, https://github.com/lunliu454/infect_place.

## Code availability

All codes for analysis and visualization presented in this manuscript is available at https://github.com/lunliu454/infect_place. This work is licensed under a Creative Commons Attribution 4.0 International (CC BY 4.0) license, which permits unrestricted use, distribution, and reproduction in any medium, provided the original work is properly cited. To view a copy of this license, visit https://creativecommons.org/licenses/by/4.0/.
